# Gels: Synthesis, Characterization and Applications in High Performance Chemistry (2nd Edition)

**DOI:** 10.3390/gels11050351

**Published:** 2025-05-10

**Authors:** Viorel-Puiu Paun, Maria-Alexandra Paun

**Affiliations:** 1 Department of Physics, Faculty of Applied Sciences, University Politehnica of Bucharest, 060042 Bucharest, Romania; viorel_paun2006@yahoo.com; 2 Academy of Romanian Scientists, 050094 Bucharest, Romania; 3 Federal Office of Communications (OFCOM), 2501 Bienne, Switzerland

This Editorial of the Special Issue proposed and managed by Prof. Viorel-Puiu Paun, Guest Editor, has been partially prepared by his daughter, Dr. Maria-Alexandra Paun, in addition to the valuable notes of her late father. The significant work, started by her father in 2023, was carried on by Dr. Maria-Alexandra Paun, Guest Editor, after the passing of Prof. Viorel-Puiu Paun in April 2024 and until the closing of this Special Issue in October 2024. Most of the papers published in this Special Issue (11 out of 15) had Prof. Viorel-Puiu Paun as Academic Editor. In memoriam of their loving father and husband, the deeply and profoundly saddened family has prepared the following obituary lines to highlight the excellent scientific and humane qualities of Prof. Viorel-Puiu Paun.

The Romanian and international research community have lost a prominent personality in the field of physics and mathematics. Prof. Viorel Puiu Paun, an outstanding university professor from the Politehnica University of Bucharest and a member of the Romanian Academy of Scientists, has passed on to the eternal. He was a remarkable father to his two children, Maria-Alexandra and Vladimir-Alexandru and husband to his wife, Jenica. On behalf of the deeply saddened family in mourning, together with my mother, Mrs. Jenica Paun, and my brother, Dr. Vladimir-Alexandru Paun, we would like to express our eternal gratitude for the most remarkable person he was, the most loving, caring, devoted, and supportive father one could ever have. We love him dearly, and he will always be with us in our memories and in our lives forever!

He dedicated his whole life first to his family and secondly to education, science, and knowledge. He was a full professor in the Physics Department of the Politehnica University of Bucharest, Faculty of Automation and Computers and Faculty of Applied Sciences, where he taught physics at the highest level and guided students in their doctoral theses.

An internationally distinguished personality and prolific scientist, renowned, appreciated, and recognized by people from all over the world, recipient of many awards and distinctions, with an impressive intellect, many challenging subjects were very easy for him as he had a level of knowledge and understanding of science that was beyond any reality; he was in an outstanding and privileged realm of the most impressive minds that ever existed in this world. His extensive education, with two master’s degrees in both mathematics and physics, two PhDs in physics and biomedical engineering, and other important training, strongly coupled with a vivacious interest in a multitude of different fields and an agile scientific curiosity, made Prof. Viorel-Puiu Paun an excellent scientist, an accomplished interdisciplinary personality, and an admired academician. In addition, he had a personal charm that made him very endearing and respected throughout the world, in academic environments of the highest levels.

His most important contributions include more than 200 scientific publications, with more than 150 articles published in reputable ISI-indexed international journals with high impact factors, and a significant number of more than 20 books and course materials. For his published research activity, he received numerous citations and held a high h-index of 24. He had a natural gift and passion for teaching, performing research, and writing scientific papers, which he carried with him until the very last day of his life. He was the first person to teach the Romanian community about the importance of publishing in ISI-indexed journals, as he led several workshops showing people how to publish and share/interpret the metrics associated with publications.

The expression of his utmost devotion to teaching and research was the fact that he continued to teach and publish until the very last moment. He was very active and remained so with numerous scientific publications and Special Issues in progress.

His publishing activity can be found on the following platforms:

(1) ResearchGate: Viorel-Puiu PAUN|Professor (Full)|PhD|University Politehnica of Bucharest, Bucharest|UPB|Department of Physics|Research profile (http://www.researchgate.net/profile/Viorel-Puiu-Paun accessed on 1 May 2025).
(2) Google Scholar: Viorel-Puiu Paun‬-Google Academic (https://scholar.google.com/citations?user=59QL-XAAAAAJ&hl=ro accessed on 1 May 2025).
(3) Web of Science: Clarivate (http://www.webofscience.com/wos/ accessed on 1 May 2025).

God rest him in peace. We will always love him, and we will never forget him. Eternal gratitude and love from his family. 

With eternal love, 

Jenica, Maria-Alexandra, and Vladimir-Alexandru Paun

The present Editorial showcases the published contents of the Special Issue titled “Gels: Synthesis, Characterization, and Applications in High-Performance Chemistry (2nd Edition)”, proposed, written, and managed by Prof. Viorel-Puiu Paun, Guest Editor (and continued by Dr. Maria-Alexandra Paun, after the passing away of Prof. Viorel-Puiu Paun), in 2023–2024, which provided an innovative continuation of the first Special Issue, titled “Gels: Synthesis, Characterization and Applications in High Performance Chemistry”, also prepared, written, and managed by Prof. Viorel-Puiu Paun, Guest Editor, published in 2022–2023, which was a great success, with 16 distinct published papers plus an Editorial, signed by Prof. Viorel-Puiu Paun, and published in 2023 as a book, with more than 46,000 views at the time of the preparation of this document.

Organogels, hydrogels, and ionic gels are investigated both theoretically and experimentally. Detailed research is focused on both their synthesis and their applications in high-performance chemistry and its important branches. All the gels mentioned above are characterized from structural and supramolecular points of view via FTIR, NMR, X-ray diffraction, and POM. On this occasion, the proposed keywords, respectively, organogels, hydrogels, ionic gels, chitosan, and fractal analysis, which were considered extremely significant and valuable, were addressed in great technical detail in this innovative scientific study essay. Consequently, all these reference themes, as well as those inherently associated with them, were approached and elegantly synthesized in the Editorial.

Articles that appear in this Special Issue focus on one or more of the topics listed above. At this moment in time, with all scientific works having been published in the journal under the auspices of the Special Issue proposed by the Guest Editor, Prof. Viorel-Puiu, who later became the Editor of this volume, together with Dr. Maria-Alexandra Paun, we can state our reflection that the central topic pertains to the refined analysis of hydrogels, with an emphasis on chitosan. The employment of xerogels for different applications was also investigated through a plethora of scientific means, such as gravimetrical measurements, SEM imaging, and fractal analysis of SEM pictures. The fractal analysis of such gels was pioneered and thoroughly examined by Prof. Viorel-Puiu Paun in this Special Issue and other of his past publications. Images were scrutinized and reviewed by cleverly calculating the fractal dimension and the lacunarity as a quantitative measure of the homogeneity of the material and its texture through their topological analysis in some of the published papers. Moreover, highly interest-worthy aspects using different types of gels in reinforcement, flood prevention, ecological restoration, combustion enhancement, corrosion protection, acoustic propagation, electrochemical storage, synthesis of nanoparticles, gas sensing, ozone detection, the evaluation of coatings, the development of concrete, and obtaining magnetic nanoparticles were considered in the scientific papers included in this Special Issue.

The works edited and included in this Special Issue are the exact ones that appeared in *Gels* in the Special Issue with the same name; more precisely, it includes 15 distinct published papers in 2023–2024, out of which two are reviews, with more than 31,000 views at the time of the preparation of this document, plus an Editorial signed by the Editors of this volume. The papers are presented succinctly in continuation.

The first work presented is titled “Acoustic Fractional Propagation in Terms of Porous Xerogel and Fractal Parameters” by the authors Maria-Alexandra Paun, Vladimir-Alexandru Paun, and Viorel-Puiu Paun. This article portrays solid xerogel-type materials, based on chitosan, TEGylated phenothiazine, and TEG (tri-ethylene glycol), dotted with a large number of pores, that are effectively represented in their constitutive structure. They were assumed to be fractal geometrical entities and adjudged as such. The acoustic fractional propagation equation in a fractal porous medium was successfully applied and solved with the help of Bessel functions. In addition, the fractal character was demonstrated by the produced fractal analysis and has been proven on the evaluated scanning electron microscopy (SEM) pictures of porous xerogel compounds. The fractal parameters (more precisely, the fractal dimension), the lacunarity, and the Hurst index were calculated with great accuracy [1].

The second work presented is titled “Fractal Analysis of Four Xerogels Based on TEGylated Phenothiazine and Chitosan” by the authors Maria-Alexandra Paun, Mihai-Virgil Nichita, Vladimir-Alexandru Paun, and Viorel-Puiu Paun. The present article describes novel massive materials (in the solid phase) based on TEGylated phenothiazine and chitosan that possess great capability to recover mercury ions from constituent aqueous solutions. These were produced by chitosan hydrogelation accompanied by the formyl subsidiary item of TEGylated phenothiazine, attended by lyophilization. The delineation and structure description of the obtained material or supramolecular assembly were realized by FTIR (Fourier transform infrared) spectroscopy, X-ray diffraction, and POM (polarized light optical microscopy). The morphology of their texture was kept under observation by SEM (scanning electron microscopy). The obtained SEM images were evaluated by fractal analysis. The fractal parameters of interest were calculated, including the fractal dimension and lacunarity [2].

The third work presented is titled “Study of the Possibility of Using Sol–Gel Technology to Obtain Magnetic Nanoparticles Based on Transition Metal Ferrites” by the authors Nina Shabelskaya, Sergey Sulima, Elena Sulima, Oleg Medennikov, Marina Kulikova, Tatyana Kolesnikova, and Svetlana Sushkova. The article presents results for the magnetic nanoparticles sol–gel method synthesis of cobalt (II) ferrite and the organic–inorganic composite materials based on it. The obtained materials were characterized using X-ray phase analysis, scanning and transmission electron microscopy, and the Scherrer and Brunauer–Emmett–Teller (BET) methods. A composite materials formation mechanism was proposed, which includes a gelation stage where transition element cation chelate complexes react with citric acid and subsequently decompose under heating. The fundamental possibility of obtaining an organic–inorganic composite material based on cobalt (II) ferrite and an organic carrier using the presented method has been proven. Composite material formation was established to lead to a significant (5–9 times) increase in the sample surface area. Materials with a developed surface were formed; the surface area measured by the BET method was 83–143 m^2^/g. The resulting composite materials had sufficient magnetic properties to be mobile in a magnetic field. Consequently, wide possibilities for polyfunctional materials synthesis open up for various applications in medicine [3].

The fourth work presented is titled “Evaluation of Low-Toxic Hybrid Sol-Gel Coatings with Organic pH-Sensitive Inhibitors for Corrosion Protection of AA2024 Aluminium Alloy” by the authors Eva Jaldo Serrano, Jesús López-Sánchez, Federico García-Galván, Aida Serrano, Óscar Rodríguez de la Fuente, Violeta Barranco, Juan Carlos Galván, and Noemí Carmona. Today’s environmental needs require reductions in the weight of vehicles, thus reducing fuel consumption and associated emissions. For this reason, the use of light alloys is being studied, which, due to their reactivity, must be protected before use. In this work, the effectiveness of a hybrid sol–gel coating doped with various organic, environmentally friendly corrosion inhibitors applied to an AA2024 lightweight aluminum alloy was evaluated. Some of the inhibitors tested included pH indicators, acting as both corrosion inhibitors and optical sensors for the surface of the alloy. Samples are subjected to a corrosion test in a simulated saline environment and characterized before and after the test. The experimental results regarding their best inhibitor performance for their potential application in the transport industry were evaluated [4].

The fifth work presented is titled “Chemiresistors with In_2_O_3_ Nanostructured Sensitive Films Used for Ozone Detection at Room Temperature” by the authors Mariana Chelu, Paul Chesler, Cristian Hornoiu, Mihai Anastasescu, Jose Maria Calderon-Moreno, Daiana Mitrea, Costin Brasoveanu, Carmen Moldovan, and Mariuca Gartner. The detection of greenhouse gases is essential because harmful gases in the air diffuse rapidly over large areas in a relatively short period of time, causing air pollution that contributes to climate change with catastrophic consequences over time. Among the materials with favorable morphologies for gas detection (nanofibers, nanorods, and nanosheets), large specific surfaces, high sensitivity, and low production costs, nanostructured porous films of In_2_O_3_ obtained by the sol–gel method, deposited on alumina transducers, with gold (Au) interdigitated electrodes (IDE) and platinum (Pt) heating circuits, were selected. Sensitive films contained 10 deposited layers, involving intermediate and final thermal treatments to stabilize the sensitive film. The fabricated sensor was characterized using AFM, SEM, EDX, and XRD. The film morphology was complex, containing fibrillar formations and some quasi-spherical conglomerates. The deposited sensitive films were rough, thus favoring gas adsorption. Ozone sensing tests were performed at different temperatures. The highest response of the ozone sensor was recorded at room temperature, considered to be the working temperature for this specific sensor [5].

The sixth work (a review) is titled “An Elucidative Review of the Nanomaterial Effect on the Durability and Calcium-Silicate-Hydrate (C-S-H) Gel Development of Concrete” by the authors Farqad Yousuf Al-saffar, Leong Sing Wong, and Suvash Chandra Paul. Concrete as a building material is susceptible to degradation by environmental threats such as thermal diffusion, acid and sulphate infiltration, and chloride penetration. Hence, the inclusion of nanomaterials in concrete has a positive effect in terms of promoting its mechanical strength and durability performance, as well as resulting in energy savings due to reduced cement consumption in concrete production. This review article discusses the novel advances in research regarding C-S-H gel promotion and concrete durability improvement using nanomaterials. In summary, this review deals with topics relevant to the influence of nanomaterials on concrete’s resistance to heat, acid, sulphate, chlorides, and wear deterioration, as well as the impact on concrete microstructure and chemical bonding. The significance of this review is a critical discussion on the cementation mechanism of nanoparticles in enhancing durability properties owing to their nanofiller effect, pozzolanic reactivity, and nucleation effect. The utilization of nanoparticles enhanced the hydrolysis of cement, leading to a rise in the production of C-S-H gel. Consequently, this improvement in concrete microstructure led to a reduction in the number of capillary pores and pore connectivity, thereby improving the concrete’s water resistance. Microstructural and chemical evidence obtained using SEM and XRD indicated that nanomaterials facilitated the formation of cement gel either by reacting pozzolanically with portlandite to generate more C-S-H gel or by functioning as nucleation sites. Due to an increased rate of C-S-H gel formation, concrete enhanced with nanoparticles exhibited greater durability against heat damage, external attack by acids and sulphates, chloride diffusion, and surface abrasion. The durability improvement following nanomaterial incorporation into concrete can be summarized as enhanced residual mechanical strength, reduced concrete mass loss, reduced coefficients for thermal and chloride diffusion, improved performance against sulphates and acid attack, and increased surface resistance to abrasion [6].

The seventh work presented is titled “Layered Sol–Gel Deposition of a Sn, Ti, Zn, and Pr Mixed Oxide Thin Film with Electrical Properties for Gas Sensing” by the authors Izabella Dascalu, Cristian Hornoiu, Jose Maria Calderon Moreno, Petre Osiceanu, and Simona Somacescu. This article presents a layered mixed oxide thin film composed of Sn, Ti, Zn, and Pr obtained by sol–gel deposition for gas sensing applications. The film was characterized by X-ray diffraction (XRD), X-ray photoelectron spectroscopy (XPS), Raman spectroscopy, UV–Vis spectroscopy, scanning electron microscopy with energy dispersive X-ray spectroscopy (SEM-EDX), and electrochemical impedance spectroscopy (EIS). X-ray diffraction results showed the presence of a single crystalline phase with a cassiterite-like structure. Raman spectroscopy revealed characteristic bands of oxygen-deficient SnO_2_-based nanocrystallites. The band gap energy calculated from UV–Vis spectroscopy was Eg = 3.83 eV. The XPS proved their presence on the surface of all elements introduced by the inorganic precursors, as well as their oxidation states. Thus, Sn^4+^, Ti^4+^, Zn^2+^, and Pr^3+^ were detected on the surface. Moreover, by XPS, we highlighted the presence of OH groups and water adsorbed on the surface. SEM showed the five-layer morphology of the film after five successive depositions. Electrochemical properties were determined by EIS-impedance spectroscopy. The selectivity for gas sensing was also investigated for methane, propane, and formaldehyde, and the gas sensing mechanism was explained. The results indicated that the mixed oxide thin film exhibited high sensitivity and selectivity towards specific gases [7].

The eighth work presented is titled “A SEM-EDX Study on the Structure of Phenyl Phosphinic Hybrids Containing Boron and Zirconium” by the authors Petru Merghes, Narcis Varan, Gheorghe Ilia, Iosif Hulka, and Vasile Simulescu. The SEM-EDX method was used to investigate the structure and morphology of organic–inorganic hybrids containing zirconium, boron, and phosphorus compounds synthesized by the sol–gel method. We started by using, for the first time together, zirconyl chloride hexa-hydrate (ZrOCl_2_·6H_2_O), phenyl phosphinic acid, and triethyl borate as precursors and reagents at different molar ratios. The obtained hybrids showed a very high thermal stability and were not soluble in water or in organic solvents. As a consequence, such hybrid solid materials are suitable for applications at high temperatures. The obtained hybrids had complex 3D structures and formed organic–inorganic networks containing Zr-O-Zr, Zr-O-P, and Zr-O-B bridges. Such organic–inorganic networks are also expected to form supramolecular structures and to have many potential applications in different fields of great interest, such as catalysis, medicine, agriculture, energy storage, fuel cells, sensors, electrochemical devices, and supramolecular chemistry [8].

The ninth work presented is titled “Synthesis of Highly Luminescent Silica-Coated Upconversion Nanoparticles from Lanthanide Oxides or Nitrates Using Co-Precipitation and Sol–Gel Methods” by the authors Ana Iglesias-Mejuto, Alyne Lamy-Mendes, João Pina, Benilde F. O. Costa, Carlos A. García-González, and Luisa Durães. Upconversion nanoparticles (UCNPs) are under consideration for their use as bioimaging probes with enhanced optical performance for real-time follow-up under non-invasive conditions. Photostable and core-shell NaYF_4_:Yb^3+^, Er^3+^-SiO_2_ UCNPs obtained by a novel and simple co-precipitation method from lanthanide nitrates or oxides were herein synthesized for the first time. The sol–gel Stöber method followed by oven or supercritical gel drying was used to confer biocompatible surface properties to UCNPs by the formation of an ultrathin silica coating. Upconversion (UC) spectra were studied to evaluate the fluorescence of UCNPs upon red/near-infrared (NIR) irradiation. ζ-potential measurements, TEM analyses, XRD patterns, and long-term physicochemical stability were also assessed and confirmed that the UCNPs co-precipitation synthesis is a shape- and phase-controlling approach. The bio- and hemocompatibility of the UCNPs formulation with the highest fluorescence intensity was evaluated with murine fibroblasts and human blood, respectively, and provided excellent results that endorse the efficacy of the silica gel coating. The herein synthesized UCNPs can be regarded as efficient fluorescent probes for bioimaging purposes with the high luminescence, physicochemical stability, and biocompatibility required for biomedical applications [9].

The tenth work presented is titled “Electrochemical Storage Behavior of a High-Capacity Mg-Doped P2-Type Na_2/3_Fe_1−y_Mn_y_O_2_ Cathode Material Synthesized by a Sol–Gel Method” by the authors Mobinul Islam, Md. Shahriar Ahmed, Daseul Han, Gazi A. K. M. Rafiqul Bari, and Kyung-Wan Nam. Grid-scale energy storage applications can benefit from rechargeable sodium-ion batteries. As a potential material for making non-cobalt, nickel-free, cost-effective cathodes, Earth-abundant Na_2/3_Fe_1/2_Mn_1/2_O_2_ is of particular interest. However, Mn^3+^ ions are particularly susceptible to the Jahn–Teller effect, which can lead to an unstable structure and continuous capacity degradation. Modifying the crystal structure by aliovalent doping is considered an effective strategy to alleviate the Jahn–Teller effect. Using a sol–gel synthesis route followed by heat treatment, we succeeded in preparing an Mg-doped Na_2/3_Fe_1−y_Mn_y_O_2_ cathode. Its electrochemical properties and charge compensation mechanism were then studied using synchrotron-based X-ray absorption spectroscopy and in situ X-ray diffraction techniques. The results revealed that Mg doping reduced the number of Mn^3+^ Jahn–Teller centers and alleviated high-voltage phase transition. However, Mg doping was unable to suppress the P2–P’2 phase transition at a low-voltage discharge. An initial discharge capacity of about 196 mAh g^−1^ was obtained at a current density of 20 mAh g^−1^, and 60% of rate capability was maintained at a current density of 200 mAh g^−1^ in a voltage range of 1.5–4.3 V. This study will greatly contribute to the ongoing search for advanced and efficient cathodes from Earth-abundant elements for rechargeable sodium-ion batteries operable at room temperature [10].

The eleventh work presented is titled “Combustion Enhancement of Gel Propellant Containing High Concentration Aluminum Particles Based on Carbon Synergistic Effect” by the authors Jiyuan Chen, Hui Zhao, Weifeng Li, and Haifeng Liu. The addition of aluminum particles to gel propellants can improve combustion performance. However, the agglomeration of aluminum during the combustion process can result in a series of negative effects. In this paper, the aluminum agglomeration inhibition method of gel propellant based on the carbon synergistic effect is proposed. Carbon particles exhibit excellent combustion properties, and the gaseous product CO_2_ generated during combustion can mitigate the agglomeration of aluminum. The research demonstrates that incorporating carbon particles into an aluminum-containing gel effectively reduces the incomplete combustion of the aluminum particles and increases the volumetric calorific value of the gel. When the mass fraction of carbon is 5 wt.%, the volume calorific value of the gel reaches its highest. Meanwhile, the rheological experiments show that the addition of carbon particles can improve the shear-thinning properties of the gel, which is beneficial to the atomization and combustion processes of the gel [11].

The twelfth work presented is titled “Eco-Friendly Sol–Gel Coatings with Organic Corrosion Inhibitors for Lightweight AZ61 Alloy” by the authors Jorge Domínguez-Martínez, Jesús López-Sánchez, Federico García-Galván, Aída Serrano, Violeta Barranco, Juan Carlos Galván, Óscar Rodríguez de la Fuente, and Noemí Carmona. The latest advances in technology and materials science have catalyzed a transformative shift towards the adoption of environmentally conscious and lightweight materials across key sectors, such as the aeronautics, biomedical, and automotive industries. Noteworthy among these innovations are the magnesium–aluminum (Mg-Al) alloys employed in aeronautical applications, contributing to the overall reduction in aircraft weight and subsequently diminishing fuel consumption and mitigating atmospheric emissions. The present work delves into a study of the anti-corrosive properties inherent in various sol–gel coatings, leveraging a range of environmentally friendly corrosion inhibitors specifically tailored for samples of the AZ61 alloy. Methodologically, the work involves the synthesis and application of sol–gel coatings on AZ61 alloy containing eco-friendly inhibitors: l-cysteine, *N*-acetyl-cysteine, curcumin, and methylene blue. Subsequently, an accelerated corrosion test in a simulated saline environment was performed. Through microstructural and compositional analyses, the best inhibitor responses were achieved with inhibitors containing S and N heteroatoms and conjugated double bonds in their structure, probably due to the creation of a continuous MgCl_2_ layer. This research contributes to the ongoing discourse on protective eco-coatings, aligning with the broader paradigm shift towards sustainable and lightweight materials in key industries [12].

The thirteenth work (a review) presented is titled “Green and Low-Cost Modified Pisha Sandstone Geopolymer Gel Materials for Ecological Restoration: A Phase Review” by the authors Changming Li, Yubing Fu, Haifeng Cheng, Yaozong Wang, Dongyang Jia, and Hui Liu. Pisha sandstone (PS) is a special interbedded rock in the middle reaches of the Yellow River that experiences severe weathering and is loose and broken. Due to severe multiple erosion events, the Pisha sandstone region is called “the most severe water loss and soil erosion in the world” and “the ecological cancer of the earth”. As a special pozzolanic mineral, PS has the potential to be used as a precursor for the synthesis of green and low-carbon geopolymer gel materials and applied in ecological restoration. This paper aimed to undertake a phase review of the precursors for geopolymer gel materials. The genesis and distribution, physical and chemical characterization, erosion characteristics, and advances in the ecological restoration of PS are all summarized. Furthermore, current advances in the use of PS for the synthesis of geopolymer gel materials in terms of mechanical properties and durability are discussed. The production of Pisha sandstone geopolymer gels through the binder jetting technique and 3D printing techniques is prospected. Meanwhile, the prospects for the resource application of PS in mine rehabilitation and sustainable ecology are discussed. In the future, multifactor-driven comprehensive measures should be further investigated in order to achieve ecological restoration in the Pisha sandstone region and promote high-quality development in the Yellow River Basin [13].

The fourteenth work presented is titled “Development of Environmentally Friendly and Economical Flood-Prevention Stones Based on the Sediments of the Yellow River”, by the authors Ying Liu, Hao Xiao, Yongxiang Jia, Yajun Lv, Li Dai, and Chen Yang. The deposition of Yellow River sediment in the middle and lower reaches is a significant factor in the siltation of reservoirs and the occurrence of serious flooding along the river. The efficient and valuable utilization of Yellow River sediment has already become a key research topic in this field. In this study, Yellow River sediment was employed as the primary material, in conjunction with commercially available slag, fly ash, and quicklime as the binder, to develop a novel type of artificial flood-prevention stone. Following a 28-day standard curing procedure, the highest compressive strength of the prepared artificial stone was recorded at 4.29 MPa, with a value exceeding 0.7 MPa under wet conditions. The results demonstrated that the prepared artificial stone met the specifications for artificial flood-prevention stones. The curing mechanism, as evidenced by analyses from SEM and XRD testing, indicated that the alkali excitation process in the binder, which produced C-A-S-H gel, was the key factor in enhancing the compressive strength of the specimens. Notably, an evaluation of the amount of CO_2_ emissions and the cost of the artificial stone concluded that the preparation process was both environmentally friendly and cost-effective [14].

The fifteenth and last work presented is titled “Sol–Gel Derived Alumina Particles for the Reinforcement of Copper Films on Brass Substrates”, by the authors Samah Sasi Maoloud Mohamed, Marija M. Vuksanović, Dana G. Vasiljević-Radović, Ljiljana Janković Mandić, Radmila M. Jančić Heinneman, Aleksandar D. Marinković, and Ivana O. Mladenović. The aim of this study was to provide tailored alumina particles suitable for reinforcing the metal matrix film. The sol–gel method was chosen to prepare submicron-sized particles and to control crystal structure by calcination. In this study, copper-based metal matrix composite (MMC) films were developed on brass substrates with different electrodeposition times and alumina concentrations. Scanning electron microscopy (FE-SEM), with energy-dispersive spectroscopy (EDS), TEM, and X-ray diffraction (XRD), was used to characterize the reinforcing phase. The MMC Cu-Al_2_O_3_ films were synthesized electrochemically using the co-electrodeposition method. Microstructural and topographical analyses of pure (alumina-free) Cu films and the Cu films with incorporated Al_2_O_3_ particles were performed using FE-SEM/EDS and AFM, respectively. Hardness and adhesion resistance were investigated using the Vickers microindentation test and evaluated by applying the Chen–Gao (C-G) mathematical model. The sessile drop method was used for measuring contact angles for water. The microhardness and adhesion of the MMC Cu-Al_2_O_3_ films were improved when Al_2_O_3_ was added. The concentration of alumina particles in the electrolyte correlates with an increase in absolute film hardness in the way that 1.0 wt.% of alumina in electrolytes results in a 9.96% increase compared to the pure copper film, and the improvement is maximal in the film obtained from electrolytes containing 3.0 wt.% alumina, giving the film 2.128 GPa, a 134% hardness value similar to that of the pure copper film. The surface roughness of the MMC film increased from 2.8 to 6.9 times compared to the Cu film without particles. The decrease in the water contact angle of Cu films with incorporated alumina particles relative to the pure Cu films was from 84.94° to 58.78° [15].

Finally, we would like to express our deep appreciation and gratitude that the authors of the papers/chapters of this book, fifteen works in total, who undertook the efforts of highlighting the remarkable applications of gels in pharmaceutical uses, industry-related endeavors, ecology, chemistry, and imaging and sensing techniques aiming to facilitate and ameliorate the quality of life for people in our modern society.
